# Early Response of Radish to Heat Stress by Strand-Specific Transcriptome and miRNA Analysis

**DOI:** 10.3390/ijms20133321

**Published:** 2019-07-06

**Authors:** Zhuang Yang, Wen Li, Xiao Su, Pingfei Ge, Yan Zhou, Yuanyuan Hao, Huangying Shu, Chonglun Gao, Shanhan Cheng, Guopeng Zhu, Zhiwei Wang

**Affiliations:** Hainan Key Laboratory for Sustainable Utilization of Tropical Bioresources, College of Horticulture, Hainan University, Haikou 570228, China

**Keywords:** radish, heat stress, transcriptome sequencing, lncRNA, miRNA, physiological response

## Abstract

Radish is a crucial vegetable crop of the *Brassicaceae* family with many varieties and large cultivated area in China. Radish is a cool season crop, and there are only a few heat tolerant radish varieties in practical production with little information concerning the related genes in response to heat stress. In this work, some physiological parameter changes of young leaves under short-term heat stress were detected. Furthermore, we acquired 1802 differentially expressed mRNAs (including encoding some heat shock proteins, heat shock factor and heat shock-related transcription factors), 169 differentially expressed lncRNAs and three differentially expressed circRNAs (novel_circ_0000265, novel_circ_0000325 and novel_circ_0000315) through strand-specific RNA sequencing technology. We also found 10 differentially expressed miRNAs (ath-miR159b-3p, athmiR159c, ath-miR398a-3p, athmiR398b-3p, ath-miR165a-5p, ath-miR169g-3p, novel_86, novel_107, novel_21 and ath-miR171b-3p) by small RNA sequencing technology. Through function prediction and enrichment analysis, our results suggested that the significantly possible pathways/complexes related to heat stress in radish leaves were circadian rhythm-plant, photosynthesis—antenna proteins, photosynthesis, carbon fixation in photosynthetic organisms, arginine and proline metabolism, oxidative phosphorylation, peroxisome and plant hormone signal transduction. Besides, we identified one lncRNA–miRNA–mRNAs combination responsive to heat stress. These results will be helpful for further illustration of molecular regulation networks of how radish responds to heat stress.

## 1. Introduction

Due to the significant increase in greenhouse gas emissions from human activities, especially the oxides of carbon dioxide, methane, chlorofluorocarbons and nitrogen, the global warming and the rising temperature have made plants in danger of high-temperature stress [1]. When the ambient temperature is higher than the suitable growth temperature of plants, some catalytic enzymes in the plant slowly lose their activity, resulting in structural damage of cells, which may lead to abnormal growth, flowering and seed yield reduction [2,3].

As sessile organisms, plants have evolved lots of complex regulatory mechanisms to cope with environmental stresses [4,5]. The excess production of singlet oxygen, superoxide anion radical, hydrogen peroxide and hydroxyl, also known as reactive oxygen species (ROS), are induced in plants under abiotic stress, which undermines chloroplasts and even results in cell death [6,7]. To scavenge superfluous ROS, plants have evolved antioxidative enzymes system, including superoxide dismutase (SOD) and peroxidases (POD). Overexpression of SOD in plants can protect the physiological processes of plants by clearing superoxide. The hydrogen peroxide could be eliminated by a variety of POD [8,9]. In addition, membrane lipid peroxidation occurs under heat stress (HS), and malondialdehyde (MDA) is produced. The accumulation of osmotic adjustment substances enhances tolerance to HS as one of the physiological basis of plant heat tolerance. Plant osmotic adjustment substances mainly include amino acids, soluble sugars, soluble proteins and soluble phenols [10]. These studies suggest that the changes in soluble sugar, chlorophyll, free proline and MDA contents, as well as SOD and POD activities under HS, can be used as indicators for the evaluation and screening of crop heat tolerance.

RNA sequencing (RNA-Seq) technique is an effective and vigorous method to investigate potential functional genes and regulatory networks of plant tolerance to various stresses [11,12]. For example, Li et al [5] validated that BRs played a vital role in inducing pepper tolerance to chilling stress at the transcription level. In elite rice, several heat shock factors (HSFs) were identified after heat treatment [13].

Radish (*Raphanus sativus* L.) is an important vegetable crop of the *Brassicaceae* family including many economically important species with a low temperature optimum. It is also used for medicine. Although there are many varieties of radish in China, there is a lack of good varieties suitable for summer growth, which greatly restricts the planting range of radish and the supply of radish in summer. Therefore, studying the response mechanism of radish under heat stress and breeding heat-tolerant cultivars are of great significance.

Recently, the draft genome sequences of radish have been constructed [14], and its genome characteristics and root transcriptome have been reported [15], providing an important condition for analyzing the molecular response of radish to biotic and abiotic stresses including heat shock. Wang et al. [16] identified 6600 differentially expressed genes (DEGs) and three significant pathways in relieving heat shock-induced damages and increasing thermotolerance in radish root under heat shock. Karanja et al. [17] found 172 RsNACs in the radish genome, some of which could be involved in the response to various abiotic stresses. More and more studies have shown that non-coding RNAs (ncRNAs; including lncRNAs, miRNAs and circRNAs) play important roles in organisms [18], and the expression of some genes responsive to stress is regulated by ncRNAs [19,20]. However, the expression profiles of heat-responsive mRNA and ncRNA in radish leaves remain unclear. In this work, we obtained the data of soluble sugar, chlorophyll, free proline and MDA contents as well as SOD and POD activities, and transcriptome including mRNA, non-coding RNA from radish leaves under high temperature stress. The results would provide valuable clues for deep molecular analysis of radish heat response.

## 2. Results

### 2.1. Morphological Changes Under Heat Stress

**Figure 1:**
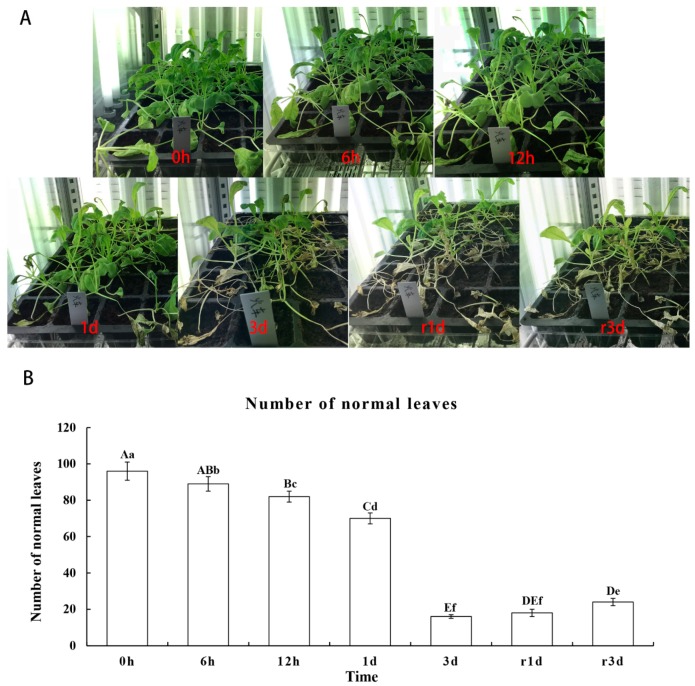
Morphological changes and normal leaf number of radish seedlings under heat stress and recovery treatment. (**A**) The radish seedlings under heat shock for 0 h, 6 h, 12 h, 1 d and 3 d as well as under recovery treatment for 1 d and 3 d. (**B**) The number of normal leaves in “Huoche” under heat shock for 0 h, 6 h, 12 h, 1 d and 3 d as well as under recovery treatment for 1 d and 3 d. Bars with different letters above the columns of figures indicate significant differences at *p* less than 0.05 (lowercase letters) or less than 0.01 (capital letters).

At 40 °C, all seedlings from the cultivar “Huoche” grew well at the initial stage, and then some of them shriveled, withered or died. After recovery treatment for 3 d, the damaged seedlings gradually returned to normal growth. These morphological data indicate that the cultivar “Huoche” has a certain heat tolerance (Figure 1).

### 2.2. Physiological Response to Heat Stress in Leaves Of The Cultivar “Huoche”

**Figure 2:**
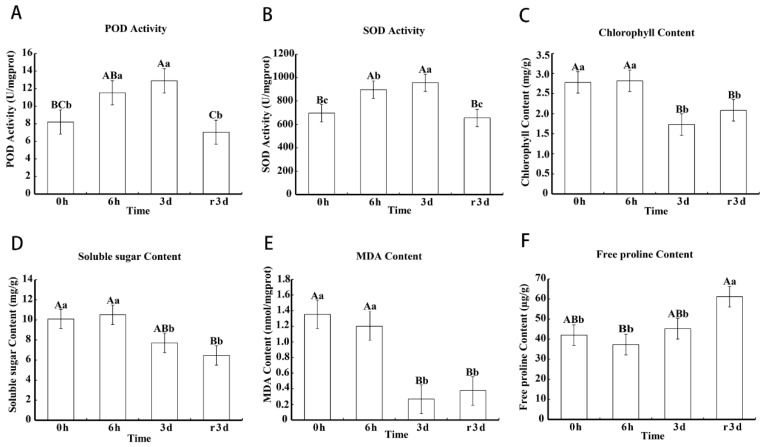
Physiological changes of radish seedlings under heat stress. (**A**–**F**) The changes in the activities of peroxidases (POD) and superoxide dismutase (SOD), the contents of chlorophyll, soluble sugar, MDA and free proline of the samples in response to heat stress (HS), respectively. Bars with different letters above the columns of figures indicate significant differences at *p* less than 0.05 (lowercase letters) or less than 0.01 (capital letters).

POD and SOD activity increased significantly after 6 h of heat stress, and still remained high after 3 d of heat stress. The heat-induced POD and SOD activity declined to control level after recovery (Figure 2A,B). Chlorophyll, soluble sugar and MDA contents were not affected by short-term heat stress, but decreased significantly after 3 d of heat stress, even during the recovery period (Figure 2C–E). We observed that heat stress did not significantly change the content of free proline (Figure 2F). These data suggest that heat stress reduces photosynthesis, but alleviates oxidative damage by increasing antioxidant enzymes in the cultivar “Huoche”.

### 2.3. Mapping and Quantitative Assessment of Illumina Sequence

To comprehensively evaluate how HS influenced transcript profile, six strand-specific RNA libraries (detecting mRNA, lncRNA and circRNA) and six small RNA libraries were constructed. For strand-specific RNA sequencing, the numbers of raw reads of these six samples range from 87.47 to 103.85 million, yielding 12.62 to 15.14 G clean bases for RNA-Seq with a Q20 percentage over 98.06%, Q30 percentage over 94.83%, and a GC percentage between 42.26% and 43.25% (Table S1). For small RNA sequencing, the numbers of raw reads of these six samples range from 13.58 to 14.90 million, yielding 0.679 to 0.745G clean bases for RNA-Seq with a Q20 percentage over 97.73%, Q30 percentage over 94.87%, and a GC percentage between 49.83% and 50.13% (Table S2).

Each library was aligned with the released reference genome as mentioned above. For strand-specific RNA sequencing, over 91.82% clean reads were successfully mapped to the reference genome, in which at least 84.51% and 4.39% clean reads were uniquely mapped and multiple mapped, respectively (Table S3). For small RNA sequencing, over 84.97% reads were successfully mapped to the reference genome, in which at least 61.07% and 22.42% reads were mapped to chains with the same direction of reference sequences and chains with the opposite direction of reference sequences, respectively (Table S4).

### 2.4. Analysis of DE mRNAs

**Figure 3:**
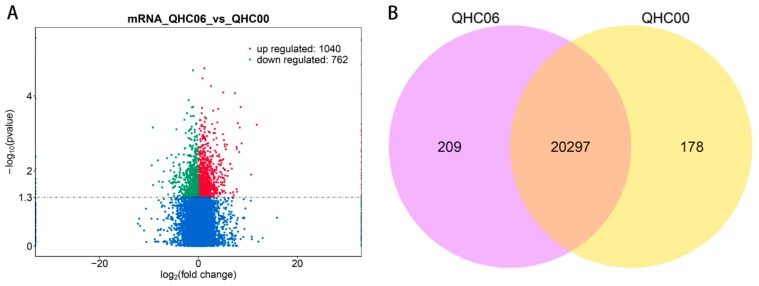
Analysis of mRNA between QHC06 and QHC00. (**A**) Volcano plot of mRNAs in QHC06 vs. QHC00. Significantly differentially expressed mRNAs are represented by red dots (up-regulated) and green dots (down-regulated), whereas non-differentially expressed mRNAs are represented by blue dots. (**B**) Venn diagram of the numbers of expressed mRNAs between QHC06 and QHC00.

In QHC06vsQHC00, 1802 differentially expressed (DE) mRNAs (1040 up-regulated and 762 down-regulated) were detected (Figure 3A, Table S5). In the meanwhile, we constructed and analyzed the Venn diagram to compare overlapping relationships between two groups (Figure 3B), and the results indicated that most mRNAs were shared by QHC00 and QHC06.

### 2.5. GO and KEGG Enrichment Analysis of DE mRNA Corresponding Genes under HS

**Figure 4:**
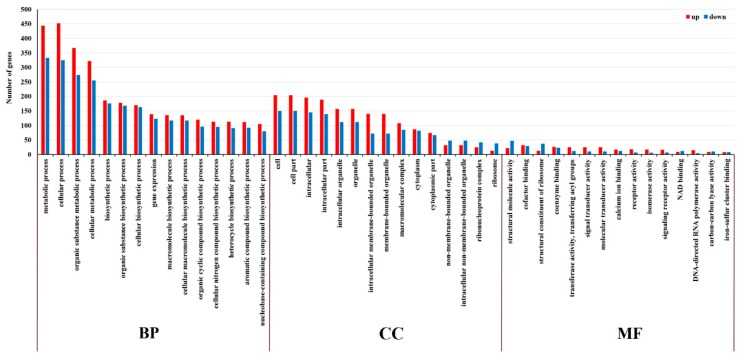
Gene ontology (GO) classification of differentially expressed (DE) mRNA corresponding genes in the cultivar “Huoche” under heat stress. The ordinate is the enriched GO term, and the abscissa is the number of differentially expressed genes in this term. Different colors are used to distinguish biological processes, cellular components, and molecular functions.

Based on gene ontology (GO) analysis of DE mRNA corresponding genes, the significantly enriched biological processes (BPs) were metabolic process (GO: 0008152), cellular process (GO: 0009987), organic substance metabolic process (GO: 0071704) and cellular metabolic process (GO: 0044237), the most significantly enriched cellular components (CCs) were cell (GO: 0005623), cell part (GO: 0044464), intracellular (GO: 0005622) and intracellular part (GO: 0044424). Structural molecule activity (GO: 0005198) and cofactor binding (GO: 0048037) were significantly enriched within the molecular function (MF) categories (Figure 4).

**Figure 5:**
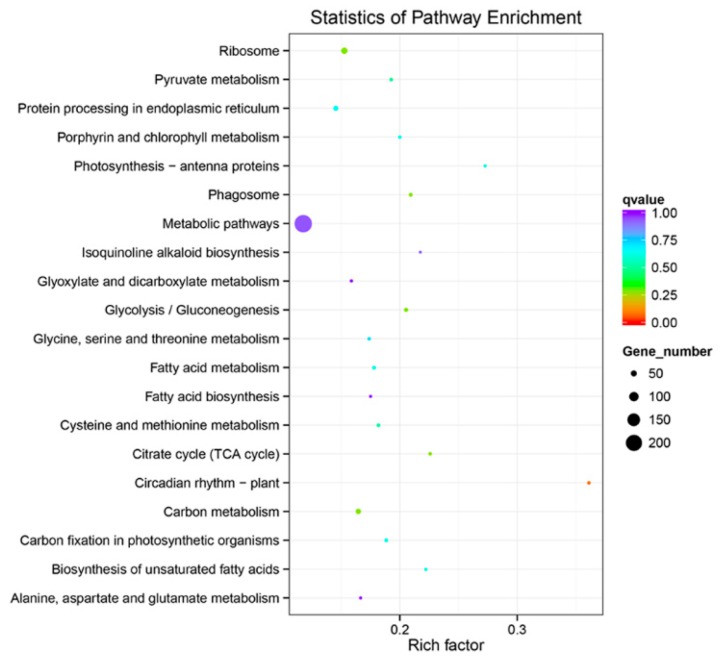
Kyoto Encyclopedia of Genes and Genome (KEGG) enrichment of DE mRNA corresponding genes in the cultivar “Huoche” under heat stress. The vertical axis represents the pathway name, and the horizontal axis represents the rich factor. The size of the point indicates the number of differentially expressed genes in the pathway, and the color of the point corresponds to a different *q*-value range.

**Figure 6:**
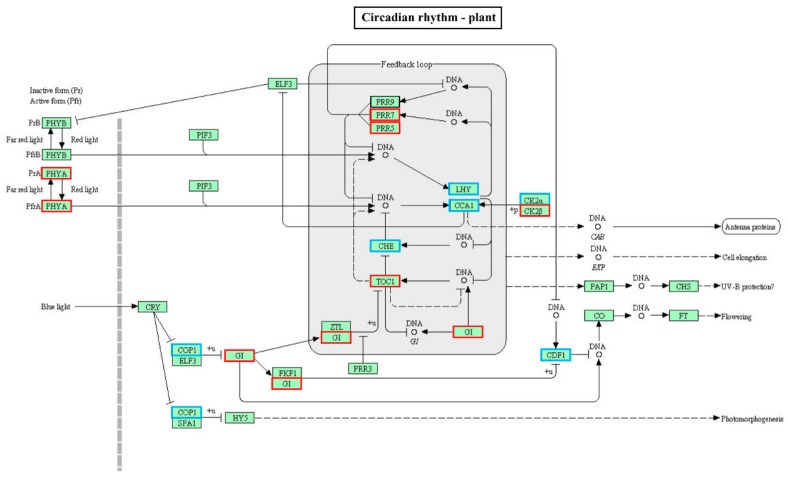
The “circadian rhythm-plant” pathway enriched by KEGG analysis of DE mRNA corresponding genes in the cultivar “Huoche” under heat stress. KEGG orthology (KO) nodes containing up-regulated genes are marked with red boxes, while KO nodes containing down-regulated genes are marked with blue boxes. PHYA, phytochrome A; COP1, E3 ubiquitin-protein ligase RFWD2; GI, gigantea; PRR7, pseudo-response regulator 7; PRR5, pseudo-response regulator 5; CHE, transcription factor TCP21 (protein CCA1 HIKING EXPEDITION); TOC1, pseudo-response regulator 1; LHY, MYB-related transcription factor LHY; CCA1, circadian clock associated 1; CK2α, casein kinase II subunit alpha; CK2β, casein kinase II subunit beta; CDF1, Dof zinc finger protein DOF5.5.

**Figure 7:**
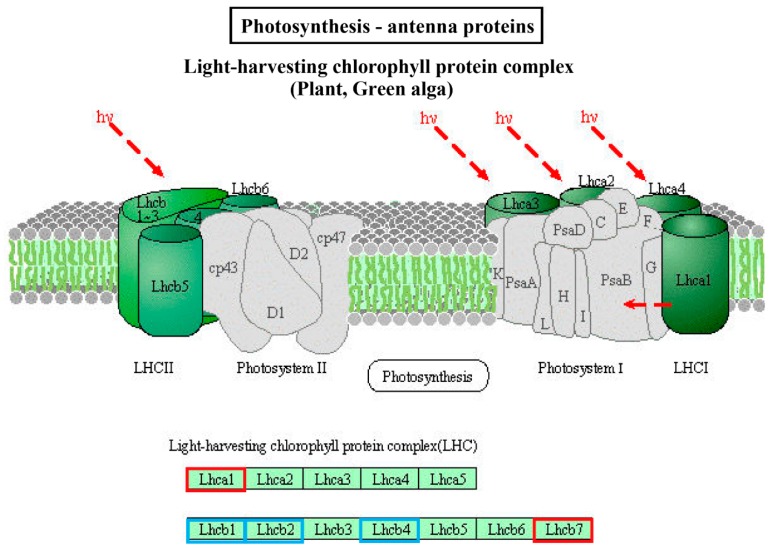
The “photosynthesis—antenna proteins” enriched by KEGG analysis of DE mRNA corresponding genes in the cultivar “Huoche” under heat stress. KO nodes containing up-regulated genes are marked with red boxes, while KO nodes containing down-regulated genes are marked with blue boxes. Lhca1, light-harvesting complex I chlorophyll a/b binding protein 1; Lhcb1, light-harvesting complex II chlorophyll a/b binding protein 1; Lhcb2, light-harvesting complex II chlorophyll a/b binding protein 2; Lhcb4, light-harvesting complex II chlorophyll a/b binding protein 4; Lhcb7, light-harvesting complex II chlorophyll a/b binding protein 7.

**Figure 8:**
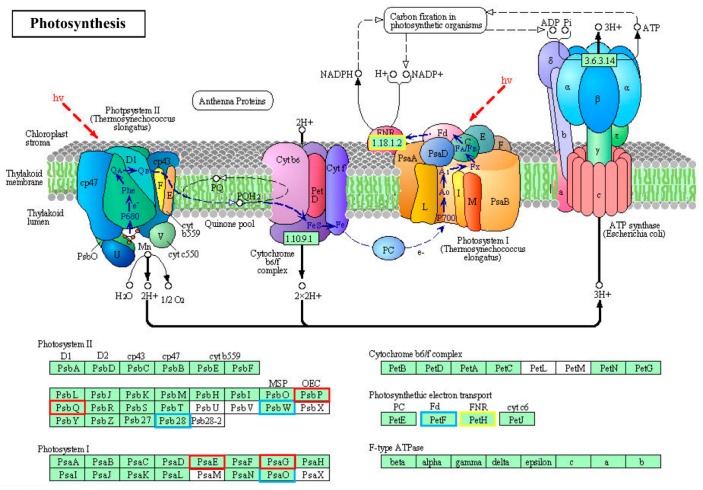
The “photosynthesis” pathway enriched by KEGG analysis of DE mRNA corresponding genes in the cultivar “Huoche” under heat stress. KO nodes containing up-regulated genes are marked with red boxes, while KO nodes containing down-regulated genes are marked with blue boxes. PsbP, photosystem II oxygen-evolving enhancer protein 2; PsbQ, photosystem II oxygen-evolving enhancer protein 3; PsbW, photosystem II PsbW protein; Psb28, photosystem II 13kDa protein; PsaE, photosystem I subunit IV; PsaG, photosystem I subunit V; PsaO, photosystem I subunit PsaO; PetF, ferredoxin.

With the Kyoto Encyclopedia of Genes and Genome (KEGG) pathway annotation, 108 pathways/ complexes were enriched (Table S6) and circadian rhythm-plant (ath04712) was notably enriched in QHC06 vs. QHC00, which might play a crucial part in the response to HS in the cultivar “Huoche” (Figure 5). In this pathway, PHYA (phytochrome A), PRR5 (pseudo-response regulator 5), PRR7 (pseudo-response regulator 7), TOC1 (pseudo-response regulator 1), CK2β (casein kinase II subunit beta) and GI (gigantea) were up-regulated, while COP1 (E3 ubiquitin-protein ligase RFWD2), CHE (transcription factor TCP21 (protein CCA1 HIKING EXPEDITION)), CCA1 (circadian clock associated 1), LHY (MYB-related transcription factor LHY), CDF1 (Dof zinc finger protein DOF5.5) and CK2α (casein kinase II subunit alpha) were down-regulated (Figure 6). Additionally, photosynthesis—antenna proteins (ath00196; Figure 7), photosynthesis (ath00195; Figure 8), carbon fixation in photosynthetic organisms (ath00710; Figure S1), arginine and proline metabolism (ath00330; Figure S2), oxidative phosphorylation (ath00190; Figure S3), peroxisome (ath04146; Figure S4) and plant hormone signal transduction (ath04075; Figure S5) could be also responsive to heat stress.

### 2.6. DE mRNAs Encoding Transcription Factor

In this study, a total of 165 DE mRNA were predicted to associate with 54 TF families (Table S7). The top four abundant types were the Orphans family with 13 DE mRNAs, the MYB family with 11 DE mRNAs, the AP2-EREBP family with 10 DE mRNAs, and the bZIP family with 10 DE mRNAs, respectively.

### 2.7. Identification of DE mRNA Encoding Heat Shock Protein (HSP) and Heat Shock Factor (HSF)

In order to further excavate the DE mRNA related to HS in the cultivar “Huoche”, we found that the heat stress transcription factor A-8-like (LOC108834589), heat shock factor-binding protein 1-like (LOC108833879), hsp70 nucleotide exchange factor fes1 (LOC108859078), hsp70 nucleotide exchange factor fes1-like (LOC108857317), 28 kDa heat- and acid-stable phosphoprotein (LOC108806935) and HEAT repeat-containing protein 5B%2C (LOC108817282) were differentially expressed (Table 1).

### 2.8. Analysis of DE lncRNAs, DE miRNAs and DE circRNAs

**Figure 9:**
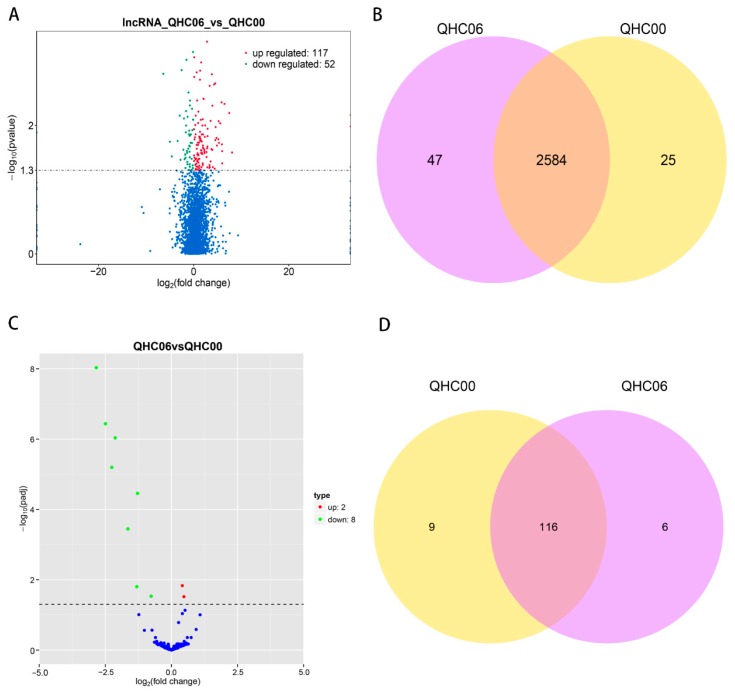
Analysis of lncRNA and miRNA between QHC06 and QHC00. (**A**) Volcano plot of lncRNAs in QHC06 vs. QHC00. Significantly differentially expressed lncRNAs are represented by red dots (up-regulated) and green dots (down-regulated), whereas non-differentially expressed lncRNAs are represented by blue dots. (**B**) Venn diagram of the numbers of expressed lncRNAs between QHC06 and QHC00. (**C**) Volcano plot of miRNAs in QHC06 vs. QHC00. Significantly differentially expressed miRNA are represented by red dots (up-regulated) and green dots (down-regulated), whereas non-differentially expressed genes were represented by blue dots. (**D**) Venn diagram of the numbers of expressed miRNAs between QHC06 and QHC00.

**Figure 10:**
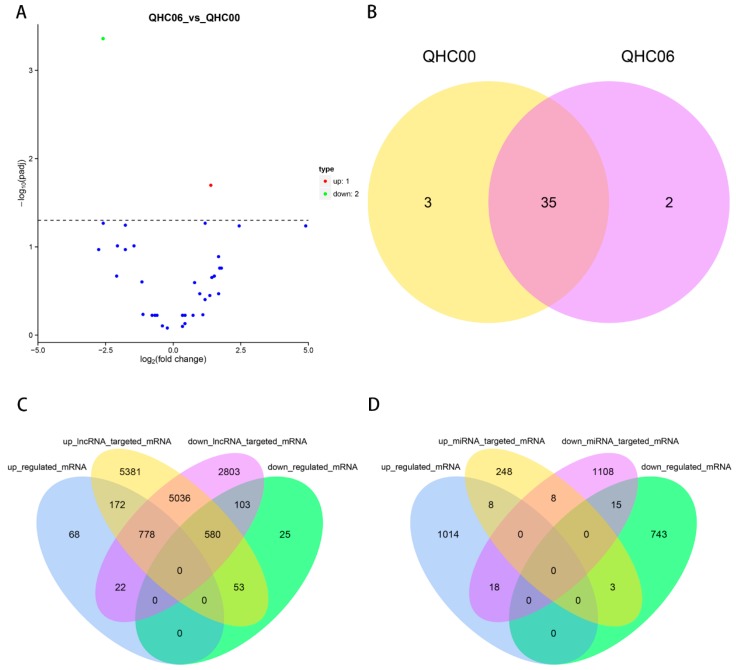
circRNA analysis and comprehensive analysis between QHC06 and QHC00. (**A**) Volcano plot of circRNAs in QHC06 vs. QHC00. Significantly differentially expressed circRNAs are represented by red dots (up-regulated) and green dots (down-regulated), whereas non-differentially expressed circRNAs are represented by blue dots. (**B**) Venn diagram of the numbers of expressed circRNAs between QHC06 and QHC00. (**C**)Venn diagram of the intersection analysis between DE lncRNA targeted mRNA and DE mRNA. (**D**) Venn diagram of the intersection analysis between DE miRNA targeted mRNA and DE mRNA.

Compared with the control group, DE lncRNAs, miRNAs and circRNAs in the heat-treated group were displayed in the forms of a Volcano plot and Venn diagram (Figure 9A–D and Figure 10A,B). The detailed data of the up-regulated and down-regulated lncRNAs between QHC06 and QHC00 are listed in Table S8. All DE miRNAs and circRNAs are listed in Table 2 and Table 3, respectively. We identified 169 DE lncRNAs (117 up-regulated and 52 down-regulated), 10 DE miRNAs (two up-regulated and eight down-regulated) and three DE circRNAs (one up-regulated and two down-regulated) between QHC06 and QHC00, respectively.

We predicted the biological function of DE lncRNA through its co-location and co-expression with protein-coding genes. Figure 10C showed the Venn diagram of the intersection analysis between DE lncRNA targeted mRNA and DE mRNA. The numbers of up-regulated targeted mRNA of up-regulated lncRNAs, down-regulated targeted mRNA of down-regulated lncRNAs, up-regulated targeted mRNA of down-regulated lncRNAs and down-regulated targeted mRNA of up-regulated lncRNAs were 172, 103, 22 and 53, respectively (Table S9). Interestingly, a heat-related gene (HEAT repeat-containing protein 5B%2C) was also found in the list of up-regulated targeted mRNAs of up-regulated lncRNAs. We took another intersection analysis between DE miRNA targeted mRNA and DE mRNA based on psRNATarget online software and identified 18 up-regulated targeted mRNAs of down-regulated miRNAs and three down-regulated targeted mRNAs of up-regulated miRNAs under HS (Figure 10D, Table 4). These results suggest that these mRNAs would be specially controlled by corresponding lncRNAs or miRNAs in the cultivar “Huoche” in response to heat stress.

### 2.9. Functional Prediction of DE lncRNA and DE miRNA in the Cultivar “Huoche”

**Figure 11:**
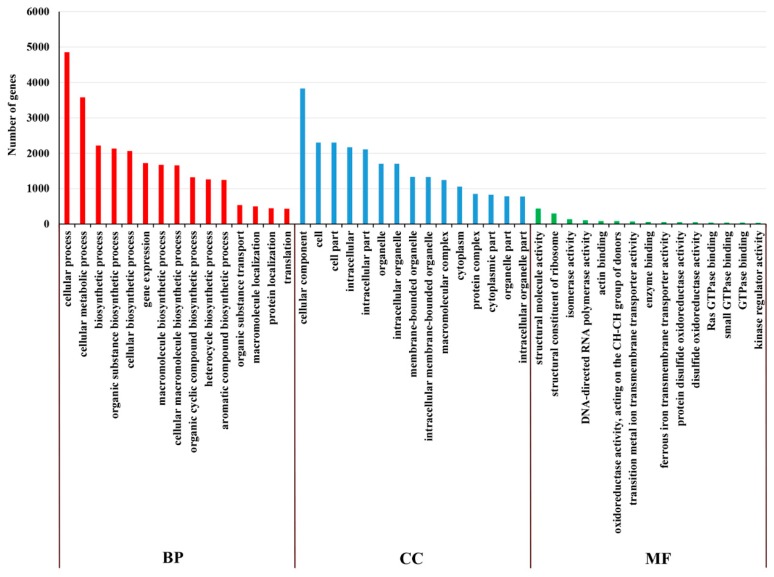
GO analysis of lncRNA in the cultivar “Huoche” under heat stress. The histogram of GO enrichment analysis of the targeted mRNA of DE lncRNAs. The ordinate is the enriched GO term, and the abscissa is the number of differentially expressed genes in this term. Different colors are used to distinguish biological processes, cellular components and molecular functions.

**Figure 12:**
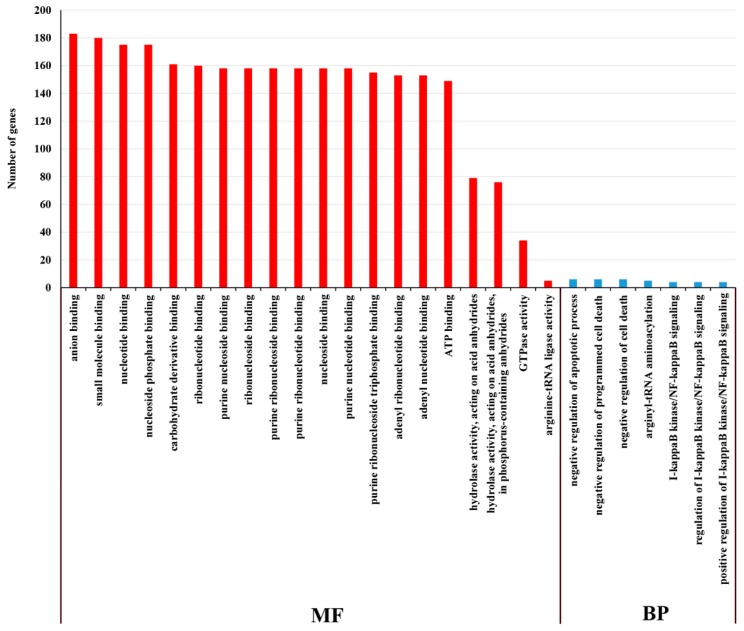
GO analysis of miRNAs in the cultivar “Huoche” under heat stress. The histogram of GO enrichment analysis of DE miRNA targeted mRNAs. The ordinate is the enriched GO term, and the abscissa is the number of differentially expressed genes in this term. Different colors are used to distinguish biological processes, cellular components and molecular functions.

On the basis of GO enrichment analysis of the targeted mRNA of DE lncRNAs, cellular process (GO: 0009987) and cellular metabolic process (GO: 0044237) were remarkably enriched among the BP categories. The most notably enriched CC was the cellular component (GO: 0005575). Among the CC categories, structural molecule activity (GO: 0005198) and structural constituent of ribosome (GO: 0003735) were significantly enriched (Figure 11). On the basis of the GO enrichment analysis of the targeted mRNA of DE miRNAs, the most remarkably enriched BPs were negative regulation of apoptotic process (GO: 0043066), negative regulation of programmed cell death (GO: 0043069) and negative regulation of cell death (GO: 0060548). Anion binding (GO: 0043168), small molecule binding (GO: 0036094), nucleotide binding (GO: 0000166) and nucleoside phosphate binding (GO: 1901265) were significantly enriched among the MF categories (Figure 12).

**Figure 13:**
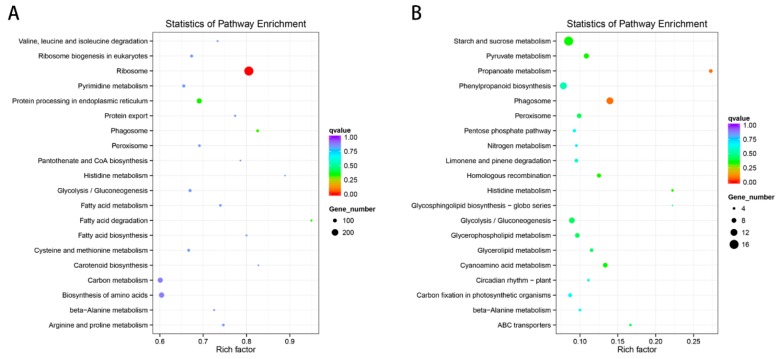
The enriched KEGG pathway scatterplots of non-coding RNAs (ncRNAs) in the cultivar “Huoche” under heat stress. (**A**) and (**B**) KEGG enrichment of lncRNAs and miRNAs in “Huoche” under HS. The vertical axis represents the pathway name, and the horizontal axis represents the Rich factor. The size of the point indicates the number of differentially expressed genes in the pathway, and the color of the point corresponds to a different *q*-value range.

We utilized KEGG enrichment analysis to determine the most important biochemical metabolic pathways and signal transduction pathways involved in specific genes. The results showed that ribosome (ath03010) was the significantly enriched KEGG pathways of DE lncRNAs (Figure 13A). The notably enriched KEGG pathways of DE miRNAs were propanoate metabolism (ath00640) and phagosome (ath04145; Figure 13B).

When comprehensively analyzing KEGG pathways among DE mRNA, DE lncRNA and DE miRNA, we found that their enriched pathways had high similarity. For instance, in the “circadian rhythm-plant” pathway, the gene in CCA1 node was enriched both in KEGG pathways of DE mRNA and DE miRNAs. The corresponding genes enriched in the “carbon fixation in photosynthetic organisms”, “arginine and proline metabolism”, “photosynthesis”, “oxidative phosphorylation”, “peroxisome” and “plant hormone signal transduction” of DE mRNA were almost enriched in the KEGG analysis of DE lncRNAs. Furthermore, the genes in EC:4.1.2.13 node and in EC:3.5.3.12 node were enriched among KEGG pathways of DE mRNA, DE lncRNA and DE miRNAs in the “carbon fixation in photosynthetic organisms” and “arginine and proline metabolism” pathways, respectively. All these results showed that these pathways might have something to do with thermotolerance of radish leaves.

### 2.10. Regulatory Network in Response to HS

After the construction of lncRNA–miRNA–mRNA combinations, we built a competing endogenous RNA (ceRNA) regulation network with XR_001945247.1 as a decoy, ath-miR165a-5p as a center, XM_018579231.1 and XM_018585227.1 as the target. Since a small number of DE circRNAs were detected, no ceRNA molecular network of circRNA–miRNA–mRNA was constructed.

## 3. Discussion

Heat stress, as a dominating global concern, has a profound effect on the crop’s growth and developments, particularly in agronomic yield [21]. In this study, we found that most of the leaves of the cultivar the cultivar “Huoche” withered and died under the heat stress of 40 °C, and some individual plants could survive. Furthermore, we detected the changes of SOD, POD and MDA under heat stress. To identify HS-related genes, we analyzed the transcriptome of young leave of the cultivar “Huoche” under HS, 1802 DE mRNAs were gained. Furthermore, some lncRNAs and miRNAs were found to be significantly induced under HS, and a regulatory combination of lncRNA–miRNA–mRNA was suggested.

### 3.1. TFs Response to HS

Transcription factors (TFs) are a group of DNA-binding proteins that regulate the expression of the relative genes, which play a pivotal part in plant tolerance to abiotic stress [22,23].

Although there are little known about their specific functions, a few Orphans transcription factors may regulate the sensitivity of heat shock [24]. MYB participates in response to heat by regulating downstream relative genes in plants [25]. AP2-EREBPs not only function as key regulators in many developmental processes but also are associated with plant responses to abiotic environmental stresses [26,27]. In plants, bZIP participates in many processes, such as stress signaling and plant stress tolerance [28]. In this study, the Orphans, MYB, AP2-EREBP and bZIP families ranked in the top four among all TFs, indicating that they may play key roles in response to HS in radish (Table S6).

Phytochrome interacting factors (PIFs) with bHLH domain have been involved in the signaling mechanism of heat response [29]. Jain et al. [30] identified that HB genes played a critical role in the development and abiotic stress response in rice. Some TCP genes could be significantly up-regulated during the early duration under heat shock in soybean [31]. The expression of *SbTCP19* was restricted by heat in *Sorghum* [32]. Zhang et al. [33] concluded that heat stress could induce the expression of *OsMSR15* in rice, and two C2H2-type zinc finger motifs were the component of *OsMSR15*. Furthermore, heat stress was able to reduce the expression and enzymatic activity of cinnamate 3-hydroxylase (C3H) [34]. In our experiment, we found that bHLH, HB, TCP, C2H2 and C3H transcription factors under HS were significantly differentially regulated, suggesting their potential role of heat tolerance in the radish cultivar “Huoche” (Table S7).

NAM, ATAF and CUC (NAC) transcription factors, a large protein family, could function by helping regulate plant abiotic stress responses, and they could be applied to enhance stress tolerance in plants by genetic engineering [35]. The overexpression of NAC TF JUNGBRUNNEN1 (JUB1; ANAC042) could promote the production of heat shock proteins and improved the thermotolerance of *Arabidopsis thaliana* [36,37]. Similarly, two NAC genes were differentially expressed were detected in our study (Table S7). Chen et al. [38] found that *OsMADS87* might help to improve the thermal resilience of rice. Duan et al. [39] identified that heat stress upregulated five *BrMADS* genes in the Chinese cabbage. We also acquired one up-regulated MADS gene (agamous-like MADS-box protein AGL19%2C) in this work (Table S7).

### 3.2. HS-Responsive HSPs and HSFs

Heat stress induces the expression of genes encoding heat shock proteins (HSPs) activated by heat shock factors (HSFs), which interact with heat shock elements in the promoter of HSP genes [40]. HSPs are a class of molecular chaperones involved in heat stress [41], including HSP100, HSP90, HSP70, HSP60 and small HSPs (sHSPs) [42]. *HSP70* is involved in the feedback control of HS and relates to the activity of *HSFA1a* [43]. Wang et al. [16] identified four up-regulated and 13 down-regulated *HSP70* transcripts under HS in radish taproots. In this study, after 6 h under HS, one up-regulated *HSP70* component (hsp70 nucleotide exchange factor fes1) and one down-regulated *HSP70* component (hsp70 nucleotide exchange factor fes1-like) were identified in radish leaves (Table 1).

HSFs play crucial roles in heat stress response by mediating the expression of HSPs [44]. On the basis of the structural features of the oligomerization domains, plant HSFs are classified into three categories, i.e., HSFA, HSFB and HSFC [43]. In tomato, *HSFA1* serves as a master regulator of induced thermotolerance [45]. *HSFA3* is controlled by the DREB2A transcription factor and is important for the establishment of heat tolerance in *Arabidopsis* [46]. *TaHsfA6f* is a transcriptional activator regulating some heat stress protection genes in wheat [47]. In the current study, *HSFA-8-like* was up-regulated after 6 h under HS in radish (Table 1). Taken together, HSPs and HSFs play a key role in the response to high temperature, and the further research of radish *HSFA-8-like* is valuable.

### 3.3. HS-Induced miRNAs 

In recent years, more and more ncRNAs have emerged as key regulatory molecules in response to high temperature, whose regulatory mechanisms have been revealed in some plants [48]. miRNAs that were predominantly 20–24 nucleotides function by silencing of target mRNAs [49].

Both Xin et al. [50] and Hivrale et al. [51] found that miR159 was up-regulated in response to HS. The overexpression of miR159 could inhibit MYB transcripts to reduce plant thermotolerance [48,52]. In our study, ath-miR159b-3p and ath-miR159c were also significantly induced under HS (Table 2). We also found that ath-miR169g-3p and ath-miR171b-3p were down-regulated in radish leaves under HS, similar to the results in *Populus tomentosa* [53,54], but miR169 and miR171 were up-regulated in response to HS in *Arabidopsis thaliana* [55,56], suggesting different regulation networks of these two small RNA under HS between *Arabidopsis* and radish. The miRNA miR398 is important for response to different abiotic stresses, especially heat stress. There are two essential target genes of miR398 in *Arabidopsis*, *CSDs* (closely related Cu/Zn-SODs) and *CCS1* (the copper chaperone for SOD) [57,58]. The CSDs could scavenge the superoxide radicals, and its expression is fine-tuned by the cleavage of miR398-directed mRNA. Under HS, CSD and CCS relate to the synthesis of HSF and HSP. Some studies have indicated that down-regulation of miR398 could up-regulate *CSDs* expression, which promotes the accumulation of HSF and HSP, helping plants resist heat stress [56,59]. In the present study, ath-miR398b-3p and ath-miR398a-3p were significantly down-regulated in radish leaves under HS (Table 2), similar to that in *Brassica rapa* and *Populus tomentosa* [54,60]. In addition, we identified that SOD activity in the radish was increased immediately after heat treatment, these results indicated that the *miR398-CSD/CCS* pathway could play a key role in response to HS in radish.

Based on the above data, there may be such an assumption for the response of radish leaf to heat stress: The chlorophyll content of radish leaves decreased under heat stress (Figure 2), and the expression of some genes in photosynthesis pathway and light capture protein complex was affected (Figure 7 and Figure 8). At the same time, circadian rhythm-plant pathway was significantly affected by heat stress, and this pathway had a direct impact on the light capture protein complex (Figure 6). Antioxidant enzymes POD and SOD increased rapidly, and membrane lipid peroxidation marker MDA decreased (Figure 2), which alleviated oxidative stress. Correspondingly, some genes of peroxisome were affected (Figure S4). In addition, plant hormone signal transduction was also significantly affected by high temperature, especially the up-regulation of ABF in ABA transduction pathway (Figure S5), which can regulate stomatal closure and alleviate heat stress. These results provided basic data for further clarifying how radish seedlings respond to heat stress at the molecular level.

## 4. Materials and Methods

### 4.1. Plant Materials and HS Treatment

The seeds of the radish cultivar “Huoche” were soaked in water at 25 °C for three days. Germinated seeds were individually sown in plastic pots (35 cm × 22 cm) containing nutrient enriched peat soil in climate chamber with 16 h light at 25 °C and 8 h dark at 15 °C, 70% humidity and 4000 l× light (PQX-330B-30HM, Ningbo Life Technology Co. Ltd., Ningbo, China). Twenty-day-old seedlings were treated by heat shock at 40 °C.

After heat treatment of 0 h (Control) and 6 h, young leaves (the second and third leaf from the top) were collected for strand-specific RNA sequencing and small RNA sequencing (labeling QHC00 and QHC06). For physiological parameter measurement, radish seedlings were heat treated (16 h light at 40 °C) and 8 h dark at 30 °C) for 3 d, and then they were treated by normal temperature (16 h light at 25 °C and 8 h dark at 15 °C) for 3 d. Three biological repeats for RNA sequencing and physiological analysis were performed, and the collected samples were frozen immediately in liquid nitrogen and stored at −80 °C for further use. QHC001, QHC002 and QHC003 represent the three biological replicates of QHC00. QHC061, QHC062 and QHC063 represent the three biological replicates of QHC06.

### 4.2. Morphological and Physiological Analysis

According to the manufacturer’s protocols (Nanjing Jiancheng Bioengineering Institute, Nanjing, China), the activities of POD, SOD and the contents of free proline, chlorophyll, soluble sugar and MDA of the samples were detected and determined using peroxidase assay kit, copper-zinc superoxide dismutase (CuZn-SOD) assay kit, proline assay kit, chlorophyll assay kit, plant soluble sugar content test kit, malondialdehyde (MDA) assay kit (TBA method), respectively.

### 4.3. RNA Isolation and RNA-Seq

The frozen samples were sent to Novogene Bioinformatics Technology Co. Ltd. (Beijing, China). Total RNA of each sample was isolated with Trizol reagents under the manufacturer’s instruction (Thermo Fisher Scientific, Shanghai, China). Afterward, RNA quantification and qualification were checked with the methods described by Zhang et al. [61].

mRNA, lncRNA and circRNA data were generated by strand-specific sequencing library using the rRNA-depleted RNA by NEBNext^®^ Ultra^TM^ Directional RNA Library Prep Kit for Illumina^®^ with the dUTP second-strand marking (NEB, Ipswich, MA, USA). Small RNA data were generated by NEBNext^®^ Multiplex Small RNA Library Prep Kit for Illumina^®^ (NEB, Ipswich, MA, USA). The detailed protocols were described previously [62,63]. All data of raw reads were uploaded in NCBI Sequence Read Archive (SRA, http://www.ncbi.nlm.nih.gov/Traces/sra/; this SRA submission will be released on 26-05-2020 or upon publication) with accession numbers of SRR8980866 (sRNA-QHC001), SRR8980865 (sRNA-QHC002), SRR8980864 (sRNA-QHC003), SRR8980863 (sRNA-QHC061), SRR8980862(sRNA-QHC062), SRR8980861 (sRNA-QHC063), SRR8980860 (lncRNA-QHC001), SRR8980859 (lncRNA-QHC002), SRR8980868 (lncRNA-QHC003), SRR8980867(lncRNA-QHC061), SRR8980858 (lncRNA-QHC062) and SRR8980857 (lncRNA-QHC063).

The clean reads were aligned with the radish reference genome (Rs1.0, https://www.ncbi.nlm.nih.gov/genome/?term=Raphanus%20sativus; 29-09-2015) using Hisat2 v2. 0.4 [64].

### 4.4. Differential Expression Analysis

DE mRNAs and DE lncRNAs were determined with the aid of the Ballgown [65]. DESeq2 was used to identified DE miRNAs and DE circRNAs [66].

### 4.5. GO and KEGG Enrichment Analysis

Gene ontology (GO) annotations and functional enrichment analysis of DE mRNA corresponding genes and DE ncRNA targeted genes were performed on the GO seq R package. GO terms are distributed into biological processes (BP), cellular components (CC) and molecular functions (MF) [67].

KEGG (Kyoto Encyclopedia of Genes and Genome) is the main public database related to the pathway. It is a systematic analysis of gene function and genomic information database, which is helpful for studying genes and expressions as a whole network (https://www.genome.jp/kegg/; 20-04-2018) [68]. By means of KOBAS v2.0 software [69], we detected the statistic enrichment of DE mRNA corresponding genes and DE ncRNA targeted genes in KEGG pathways.

### 4.6. Analysis of Transcription Factors

Transcription factors (TFs) are a group of proteins that can specifically bind to specific sequences upstream of the 5′ end of the gene, thereby ensuring that the target gene is expressed at a specific time and space at a specific intensity. Plant transcription factor prediction was implemented by iTALK 1.2 software. The basic principle is used to identify TFs by hmmscan using the TF families and rules defined in the database [70].

### 4.7. Construction of the Regulation Network of Competing Endogenous RNA

RNA can be mutually regulated by competitive binding to a common microRNA response element (MRE), which constitutes competing endogenous RNA (ceRNA). The ceRNA has been found to include protein-encoding mRNAs, non-coding RNAs and pseudogene transcripts [71,72]. Based on psRobot online version software, lncRNA–miRNA–mRNA pairs and circRNA–miRNA–mRNA pairs were screened. Cytoscape 3.7.1 software was adopted to construct the regulation networks between ncRNA and mRNA in response to heat stress.

## Figures and Tables

**Table 1:** Selected DE mRNAs related to HS in QHC06 vs. QHC00.

mRNA ID	Gene ID	Regulation	Gene Description
XM_018589933.1	108817282	up	HEAT repeat-containing protein 5B%2C
XM_018607917.1	108834589	up	heat stress transcription factor A-8-like
XM_018632926.1	108859078	up	hsp70 nucleotide exchange factor fes1
XM_018579151.1	108806935	down	28 kDa heat- and acid-stable phosphoprotein
XM_018607274.1	108833879	down	heat shock factor-binding protein 1-like
XM_018631289.1	108857317	down	hsp70 nucleotide exchange factor fes1-like

**Table 2:** The detailed information of DE miRNAs.

miRNA ID	QHC06 Read Count	QHC00 Read Count	log2 Fold Change	*p*-Value	*q*-Value	Regulation
ath-miR159b-3p	22,398.8097	16761.99	0.41194	0.00079	0.014687	up
ath-miR159c	6837.4573	4883.9258	0.4737	0.00233	0.030311	up
ath-miR398b-3p	284.819304	4018.3814	−2.8401	7.15E−11	9.30E−09	down
ath-miR398a-3p	16.1500394	158.0362	−2.4915	5.62E−09	3.65E−07	down
ath-miR165a-5p	18.7396158	111.39067	−2.1263	2.12E−08	9.20E−07	down
ath-miR169g-3p	2.63494616	22.542957	−2.2541	1.95E−07	6.35E−06	down
novel_86	28.5158186	74.079789	−1.2807	1.34E−06	3.49E−05	down
novel_107	6.19793394	24.899317	−1.6446	1.64E−05	0.000356	down
novel_21	778.011	2374.9094	−1.3037	0.00097	0.01577	down
ath-miR171b-3p	129.729447	229.73667	−0.76804	0.00204	0.029436	down

**Table 3:** The detailed information of DE circRNAs.

circRNA ID	QHC06 Read Count	QHC00 Read Count	log2 Fold Change	*p*-Value	*q*-Value	Regulation
novel_circ_0000265	202.3891602	76.29972516	1.3908	0.0015051	0.020068	up
novel_circ_0000325	7.966953075	46.82442697	−2.5937	1.09E−05	0.0004377	down
novel_circ_0000315	0	9.680244794	−6.3484	0.000834	0.016679	down

**Table 4:** Information of DE lncRNA and its DE targeted mRNA.

DE miRNA ID	miRNA Regulation	DE Targeted mRNA ID	mRNA Regulation
ath-miR171b-3p	down	XM_018577681.1	up
ath-miR165a-5p	down	XM_018579231.1	up
novel_21	down	XM_018579451.1	up
ath-miR165a-5p	down	XM_018585227.1	up
ath-miR398b-3p, ath-miR398a-3p	down	XM_018585419.1	up
ath-miR165a-5p	down	XM_018587204.1	up
ath-miR169g-3p	down	XM_018589104.1	up
novel_107	down	XM_018590932.1	up
ath-miR169g-3p	down	XM_018596526.1	up
novel_86	down	XM_018598856.1	up
ath-miR171b-3p	down	XM_018599972.1	up
ath-miR398a-3p, ath-miR398b-3p	down	XM_018604024.1	up
novel_86	down	XM_018618012.1	up
novel_107	down	XM_018620431.1	up
ath-miR171b-3p	down	XM_018622399.1	up
ath-miR398a-3p, ath-miR398b-3p	down	XM_018622487.1	up
novel_107	down	XM_018628114.1	up
novel_86	down	XM_018635962.1	up
ath-miR159c	up	XM_018609293.1	down
ath-miR159b-3p	up	XM_018617329.1	down
ath-miR159b-3p, ath-miR159c	up	XM_018634874.1	down

## Supplementary Materials

Supplementary Materials can be found online https://www.mdpi.com/1422-0067/20/13/3321/s1. **Table S1**: Quality evaluation of sample sequencing output data for strand-specific RNA libraries. **Table S2**: Quality evaluation of sample sequencing output data for small RNA libraries. **Table S3**: Reads of strand-specific RNA libraries and reference genome comparison list. **Table S4**: Reads of small RNA libraries and reference genome comparison list. **Table S5**: The detailed information of DE mRNAs. **Table S6**: KEGG analysis results of DE mRNA corresponding genes. **Table S7**: DE mRNAs encoding transcription factor. **Table S8**: The detailed information of DE lncRNAs between QHC06 and QHC00. **Table S9**: The detailed information between DE lncRNA and its DE targeted mRNA. **Figure S1**: The “carbon fixation in photosynthetic organisms” pathway enriched by KEGG analysis of DE mRNA corresponding genes in the cultivar “Huoche” under heat stress. KO nodes containing up-regulated genes are marked with red boxes, while KO nodes containing down-regulated genes are marked with blue boxes. EC:4.1.2.13, fructose-bisphosphate aldolase, class I; EC:3.1.3.11, fructose-1,6-bisphosphatase I; EC:2.7.2.3, phosphoglycerate kinase; EC:4.1.1.39, ribulose-bisphosphate carboxylase small chain; EC:4.1.1.31, phosphoenolpyruvate carboxylase. **Figure S2**: The “arginine and proline metabolism” pathway enriched by KEGG analysis of DE mRNA corresponding genes in the cultivar “Huoche” under heat stress. KO nodes containing up-regulated genes are marked with red boxes, while KO nodes containing down-regulated genes are marked with blue boxes. EC:6.3.1.2, glutamine synthetase; EC:1.4.1.4, glutamate dehydrogenase (NADP+); EC:1.2.1.38, N-acetyl-gamma-glutamyl-phosphate reductase; EC:1.14.11.2, prolyl 4-hydroxylase; EC:4.1.1.50, S-adenosylmethionine decarboxylase; EC:3.5.3.12, agmatine deiminase; EC:2.6.1.1, aspartate aminotransferase, cytoplasmic; EC:4.1.1.19, arginine decarboxylase. **Figure S3**: The “oxidative phosphorylation” pathway enriched by KEGG analysis of DE mRNA corresponding genes in the cultivar “Huoche” under heat stress. KO nodes containing up-regulated genes are marked with red boxes, while KO nodes containing down-regulated genes are marked with blue boxes. EC:3.6.1.1, inorganic pyrophosphatase; Ndufs4, NADH dehydrogenase (ubiquinone) Fe-S protein 4; Ndufs5, NADH dehydrogenase (ubiquinone) Fe-S protein 5; Ndufs6, NADH dehydrogenase (ubiquinone) Fe-S protein 6; Ndufs7, NADH dehydrogenase (ubiquinone) Fe-S protein 7; Ndufv1, NADH dehydrogenase (ubiquinone) flavoprotein 1; Ndufa2, NADH dehydrogenase (ubiquinone) 1 alpha subcomplex subunit 2; Ndufa5, NADH dehydrogenase (ubiquinone) 1 alpha subcomplex subunit 5; Ndufa6, NADH dehydrogenase (ubiquinone) 1 alpha subcomplex subunit 6; Ndufa8, NADH dehydrogenase (ubiquinone) 1 alpha subcomplex subunit 8; COX10, heme o synthase; COX17, cytochrome c oxidase assembly protein subunit 17; D, V-type H+-transporting ATPase subunit D; F, V-type H+-transporting ATPase subunit F; d, V-type H+-transporting ATPase subunit d. Figure S4: The “peroxisome” enriched by KEGG analysis of DE mRNA corresponding genes in the cultivar “Huoche” under heat stress. KO nodes containing up-regulated genes are marked with red boxes, while KO nodes containing down-regulated genes are marked with blue boxes. PXMP2, peroxisomal membrane protein 2; MPV17, protein Mpv17; ACAA1, acetyl-CoA acyltransferase 1; FAR, alcohol-forming fatty acyl-CoA reductase; AGT, alanine-glyoxylate transaminase/serine-glyoxylate transaminase/serine-pyruvate transaminase. **Figure S5**: The “plant hormone signal transduction” pathway enriched by KEGG analysis of DE mRNA corresponding genes in the cultivar “Huoche” under heat stress. KO nodes containing up-regulated genes are marked with red boxes, while KO nodes containing down-regulated genes are marked with blue boxes. AUX1, auxin influx carrier (AUX1 LAX family); ARF, auxin response factor; GH3, auxin responsive GH3 gene family; SAUR, SAUR family protein; A-ARR, two-component response regulator ARR-A family; PYR/PYL, abscisic acid receptor PYR/PYL family; PP2C, protein phosphatase 2C; ABF, ABA responsive element binding factor; EBF1/2, EIN3-binding F-box protein; EIN3, ethylene-insensitive protein 3; BSK, BR-signaling kinase; CYCD3, cyclin D3, plant; JAZ, jasmonate ZIM domain-containing protein.

## Abbreviations

ROS	Reactive oxygen species
SOD	Superoxide dismutase
POD	Peroxidases
HS	Heat stress
MDA	Malondialdehyde
RNA-Seq	RNA sequencing
DEGs	Differentially expressed genes
ncRNAs	Non-coding RNAs
r1d	Recovery treatment for 1 d
r3d	Recovery treatment for 3 d
DE	Differentially expressed
GO	Gene Ontology
BP	Biological processes
CC	Cellular components
MF	Molecular functions
KEGG	Kyoto Encyclopedia of Genes and Genome
PHYA	Phytochrome A
PRR5	Pseudo-response regulator 5
PRR7	Pseudo-response regulator 7
TOC1	Pseudo-response regulator 1
CK2β	Casein kinase II subunit beta
GI	Gigantea
COP1	E3 ubiquitin-protein ligase RFWD2
CHE	Transcription factor TCP21 (protein CCA1 HIKING EXPEDITION)
CCA1	Circadian clock associated 1
LHY	MYB-related transcription factor LHY
CDF1	Dof zinc finger protein DOF5.5
CK2α	Casein kinase II subunit alpha
KO	KEGG orthology
Lhca1	Light-harvesting complex I chlorophyll a/b binding protein 1
Lhcb1	Light-harvesting complex II chlorophyll a/b binding protein 1
Lhcb2	Light-harvesting complex II chlorophyll a/b binding protein 2
Lhcb4	Light-harvesting complex II chlorophyll a/b binding protein 4
Lhcb7	Light-harvesting complex II chlorophyll a/b binding protein 7
PsbP	Photosystem II oxygen-evolving enhancer protein 2
PsbQ	Photosystem II oxygen-evolving enhancer protein 3
PsbW	Photosystem II PsbW protein
Psb28	Photosystem II 13kDa protein
PsaE	Photosystem I subunit IV
PsaG	Photosystem I subunit V
PsaO	Photosystem I subunit PsaO
PetF	ferredoxin
TFs	Transcription factors
ceRNA	Competing endogenous RNA
HSP	Heat shock protein
HSF	Heat shock factor
PIFs	Phytochrome interacting factors
C3H	Cinnamate 3-hydroxylase
NAC	NAM, ATAF, and CUC
sHSPs	Small HSPs
CuZn-SOD	Copper-zinc superoxide dismutase
MRE	MicroRNA response element
Ndufs4	NADH dehydrogenase (ubiquinone) Fe-S protein 4
Ndufs5	NADH dehydrogenase (ubiquinone) Fe-S protein 5
Ndufs6	NADH dehydrogenase (ubiquinone) Fe-S protein 6
Ndufs7	NADH dehydrogenase (ubiquinone) Fe-S protein 7
Ndufv1	NADH dehydrogenase (ubiquinone) flavoprotein 1
Ndufa2	NADH dehydrogenase (ubiquinone) 1 alpha subcomplex subunit 2
Ndufa5	NADH dehydrogenase (ubiquinone) 1 alpha subcomplex subunit 5
Ndufa6	NADH dehydrogenase (ubiquinone) 1 alpha subcomplex subunit 6
Ndufa8	NADH dehydrogenase (ubiquinone) 1 alpha subcomplex subunit 8
COX10	Heme o synthase
COX17	Cytochrome c oxidase assembly protein subunit 17
PXMP2	Peroxisomal membrane protein 2
MPV17	Protein Mpv17
ACAA1	Acetyl-CoA acyltransferase 1
FAR	Alcohol-forming fatty acyl-CoA reductase
AGT	Alanine-glyoxylate transaminase/serine-glyoxylate transaminase/serine-pyruvate Transaminase
AUX1	Auxin influx carrier (AUX1 LAX family)
ARF	Auxin response factor
GH3	Auxin responsive GH3 gene family
SAUR	SAUR family protein
A-ARR	Two-component response regulator ARR-A family
PYR/PYL	Abscisic acid receptor PYR/PYL family
PP2C	Protein phosphatase 2C
ABF	ABA responsive element binding factor
EBF1/2	EIN3-binding F-box protein
EIN3	Ethylene-insensitive protein 3
BSK	BR-signaling kinase
CYCD3	Cyclin D3, plant
JAZ	Jasmonate ZIM domain-containing protein

## References

[1] Song L., Chow W.S., Sun L., Li C., Peng C. (2010). Acclimation of photosystem II to high temperature in two *Wedelia* species from different geographical origins: Implications for biological invasions upon global warming. J. Exp. Bot..

[2] Miller G.A.D., Mittler R.O.N. (2006). Could heat shock transcription factors function as hydrogen peroxide sensors in plants?. Ann. Bot..

[3] Kotak S., Larkindale J., Lee U., von Koskull-Döring P., Vierling E., Scharf K.D. (2007). Complexity of the heat stress response in plants. Curr. Opin. Plant Biol..

[4] Wang M., Zou Z., Li Q., Xin H., Zhu X., Chen X., Li X. (2017). Heterologous expression of three *Camellia sinensis* small heat shock protein genes confers temperature stress tolerance in yeast and *Arabidopsis thaliana*. Plant Cell Rep..

[5] Li J., Yang P., Kang J., Gan Y., Yu J., Calderón-Urrea A., Lyu J., Zhang G., Feng Z., Xie J. (2016). Transcriptome analysis of pepper (*Capsicum annuum*) revealed a role of 24-epibrassinolide in response to chilling. Front. Plant Sci..

[6] Gill S.S., Tuteja N. (2010). Reactive oxygen species and antioxidant machinery in abiotic stress tolerance in crop plants. Plant Physiol. Biochem..

[7] Anwar A., Yan Y., Liu Y., Li Y., Yu X. (2018). 5-aminolevulinic acid improves nutrient uptake and endogenous hormone accumulation, enhancing low-temperature stress tolerance in cucumbers. Int. J. Mol. Sci..

[8] Mittler R. (2002). Oxidative stress, antioxidants and stress tolerance. Trends Plant Sci..

[9] Meloni D.A., Oliva M.A., Martinez C.A., Cambraia J. (2003). Photosynthesis and activity of superoxide dismutase, peroxidase and glutathione reductase in cotton under salt stress. Environ. Exp. Bot..

[10] Chaitanya K.V., Sundar D., Reddy A.R. (2001). Mulberry leaf metabolism under high temperature stress. Biol. Plant.

[11] Marioni J.C., Mason C.E., Mane S.M., Stephens M., Gilad Y. (2008). RNA-seq: An assessment of technical reproducibility and comparison with gene expression arrays. Genome Res..

[12] Wang Z., Gerstein M., Snyder M. (2009). RNA-Seq: A revolutionary tool for transcriptomics. Nat. Rev. Genet..

[13] Fu C., Wang F., Liu W., Liu D., Li J., Zhu M., Liao Y., Liu Z., Huang H., Zeng X. (2017). Transcriptomic analysis reveals new insights into high-temperature-dependent glume-unclosing in an elite rice male sterile line. Front. Plant Sci..

[14] Kitashiba H., Li F., Hirakawa H., Kawanabe T., Zou Z., Hasegawa Y., Tonosaki K., Shirasawa S., Fukushima A., Yokoi S. (2014). Draft sequences of the radish (*Raphanus sativus* L.) genome. DNA Res..

[15] Mitsui Y., Shimomura M., Komatsu K., Namiki N., Shibata-Hatta M., Imai M., Katayose Y., Mukai Y., Kanamori H., Kurita K. (2015). The radish genome and comprehensive gene expression profile of tuberous root formation and development. Sci. Rep..

[16] Wang R., Mei Y., Xu L., Zhu X., Wang Y., Guo J., Liu L. (2018). Genome-wide characterization of differentially expressed genes provides insights into regulatory network of heat stress response in radish (*Raphanus sativus* L.). Funct. Integr. Genom..

[17] Karanja B.K., Xu L., Wang Y., Muleke E.M.m., Jabir B.M., Xie Y., Zhu X., Cheng W., Liu L. (2017). Genome-wide characterization and expression profiling of *NAC* transcription factor genes under abiotic stresses in radish (*Raphanus sativus* L.). PeerJ.

[18] Wang Z., Wu Z., Raitskin O., Sun Q., Dean C. (2014). Antisense-mediated *FLC* transcriptional repression requires the P-TEFb transcription elongation factor. Proc. Natl. Acad. Sci. USA.

[19] Contreras-Cubas C., Palomar M., Arteaga-Vázquez M., Reyes J.L., Covarrubias A.A. (2012). Non-coding RNAs in the plant response to abiotic stress. Planta.

[20] Nejat N., Mantri N. (2018). Emerging roles of long non-coding RNAs in plant response to biotic and abiotic stresses. Crit. Rev. Biotechnol..

[21] Li B., Gao K., Ren H., Tang W. (2018). Molecular mechanisms governing plant responses to high temperatures. J. Integr. Plant Biol..

[22] Nakashima K., Yamaguchi-Shinozaki K., Shinozaki K. (2014). The transcriptional regulatory network in the drought response and its crosstalk in abiotic stress responses including drought, cold, and heat. Front. Plant Sci..

[23] Essebier A., Lamprecht M., Piper M., Bodén M. (2017). Bioinformatics approaches to predict target genes from transcription factor binding data. Methods.

[24] Jones D.L., Petty J., Hoyle D.C., Hayes A., Oliver S.G., Riba-Garcia I., Gaskell S.J., Stateva L. (2004). Genome-wide analysis of the effects of heat shock on a *Saccharomyces cerevisiae* mutant with a constitutively activated cAMP-dependent pathway. Comp. Funct. Genom..

[25] Lim C.J., Yang K.A., Hong J.K., Choi J.S., Yun D.J., Hong J.C., Chung W.S., Lee S.Y., Cho M.J., Lim C.O. (2006). Gene expression profiles during heat acclimation in *Arabidopsis thaliana* suspension-culture cells. J. Plant Res..

[26] Riechmann J.L., Meyerowitz J.L. (1998). The AP2/EREBP family of plant transcription factors. Biol. Chem..

[27] Tang M., Liu X., Deng H., Shen S. (2011). Over-expression of *JcDREB*, a putative AP2/EREBP domain-containing transcription factor gene in woody biodiesel plant *Jatropha curcas*, enhances salt and freezing tolerance in transgenic *Arabidopsis thaliana*. Plant Sci..

[28] Zhao J., Zhang X., Wollenweber B., Jiang D., Liu F. (2008). Water deficits and heat shock effects on photosynthesis of a transgenic *Arabidopsis thaliana* constitutively expressing *ABP9*, a bZIP transcription factor. J. Exp. Bot..

[29] Bita C., Gerats T. (2013). Plant tolerance to high temperature in a changing environment: Scientific fundamentals and production of heat stress-tolerant crops. Front. Plant Sci..

[30] Jain M., Tyagi A.K., Khurana J.P. (2008). Genome-wide identification, classification, evolutionary expansion and expression analyses of homeobox genes in rice. FEBS J..

[31] Feng Z.J., Xu S.C., Liu N., Zhang G.W., Hu Q.Z., Gong Y.M. (2018). Soybean TCP transcription factors: Evolution, classification, protein interaction and stress and hormone responsiveness. Plant Physiol. Biochem..

[32] Francis A., Dhaka N., Bakshi M., Jung K.H., Sharma M.K., Sharma R. (2016). Comparative phylogenomic analysis provides insights into TCP gene functions in *Sorghum*. Sci. Rep..

[33] Zhang X., Zhang B., Li M.J., Yin X.M., Huang L.F., Cui Y.C., Wang M.L., Xia X. (2016). *OsMSR15* encoding a rice C2H2-type zinc finger protein confers enhanced drought tolerance in transgenic *Arabidopsis*. J. Plant Biol..

[34] Martinez V., Mestre T.C., Rubio F., Girones-Vilaplana A., Moreno D.A., Mittler R., Rivero R.M. (2016). Accumulation of flavonols over hydroxycinnamic acids favors oxidative damage protection under abiotic stress. Front. Plant Sci..

[35] Shao H., Wang H., Tang X. (2015). NAC transcription factors in plant multiple abiotic stress responses: Progress and prospects. Front. Plant Sci..

[36] Shahnejat-Bushehri S., Mueller-Roeber B., Balazadeh S. (2012). *Arabidopsis* NAC transcription factor JUNGBRUNNEN1 affects thermomemory-associated genes and enhances heat stress tolerance in primed and unprimed conditions. Plant Signal. Behav..

[37] Wu A., Allu A.D., Garapati P., Siddiqui H., Dortay H., Zanor M.-I., Asensi-Fabado M.A., Munné-Bosch S., Antonio C., Tohge T. (2012). *JUNGBRUNNEN1*, a reactive oxygen species–responsive NAC transcription factor, regulates longevity in *Arabidopsis*. Plant Cell.

[38] Chen C., Begcy K., Liu K., Folsom J.J., Wang Z., Zhang C., Walia H. (2016). Heat stress yields a unique MADS box transcription factor in determining seed size and thermal sensitivity. Plant Physiol..

[39] Duan W., Song X., Liu T., Huang Z., Ren J., Hou X., Li Y. (2015). Genome-wide analysis of the MADS-box gene family in *Brassica rapa* (Chinese cabbage). Mol. Genet. Genom..

[40] Chauhan H., Khurana N., Agarwal P., Khurana P. (2011). Heat shock factors in rice (*Oryza sativa* L.): Genome-wide expression analysis during reproductive development and abiotic stress. Mol. Genet. Genom..

[41] Wang W., Vinocur B., Shoseyov O., Altman A. (2004). Role of plant heat-shock proteins and molecular chaperones in the abiotic stress response. Trends Plant Sci..

[42] Al-Whaibi M.H. (2011). Plant heat-shock proteins: A mini review. J. King Saud Univ. Sci..

[43] Qu A.L., Ding Y.F., Jiang Q., Zhu C. (2013). Molecular mechanisms of the plant heat stress response. Biochem. Biophys. Res. Commun..

[44] Guo M., Liu J.H., Ma X., Luo D.X., Gong Z.H., Lu M.H. (2016). The plant heat stress transcription factors (hsfs): Structure, regulation, and function in response to abiotic stresses. Front. Plant Sci..

[45] Mishra S.K., Tripp J., Winkelhaus S., Tschiersch B., Theres K., Nover L., Scharf K.D. (2002). In the complex family of heat stress transcription factors, HsfA1 has a unique role as master regulator of thermotolerance in tomato. Genes Dev..

[46] Schramm F., Larkindale J., Kiehlmann E., Ganguli A., Englich G., Vierling E., Von Koskull-Döring P. (2008). A cascade of transcription factor DREB2A and heat stress transcription factor HsfA3 regulates the heat stress response of *Arabidopsis*. Plant J..

[47] Xue G.-P., Drenth J., McIntyre C.L. (2015). TaHsfA6f is a transcriptional activator that regulates a suite of heat stress protection genes in wheat (*Triticum aestivum* L.) including previously unknown Hsf targets. J. Exp. Bot..

[48] Zhao J., He Q., Chen G., Wang L., Jin B. (2016). Regulation of non-coding RNAs in heat stress responses of plants. Front. Plant Sci..

[49] Rogers K., Chen X. (2013). Biogenesis, turnover, and mode of action of plant microRNAs. Plant Cell.

[50] Xin M., Wang Y., Yao Y., Xie C., Peng H., Ni Z., Sun Q. (2010). Diverse set of microRNAs are responsive to powdery mildew infection and heat stress in wheat (*Triticum aestivum* L.). BMC Plant Biol..

[51] Hivrale V., Zheng Y., Puli C.O.R., Jagadeeswaran G., Gowdu K., Kakani V.G., Barakat A., Sunkar R. (2016). Characterization of drought- and heat-responsive microRNAs in switchgrass. Plant Sci..

[52] Reyes J.L., Chua N.H. (2007). ABA induction of miR159 controls transcript levels of two MYB factors during *Arabidopsis* seed germination. Plant J..

[53] Lu S., Sun Y.H., Chiang V.L. (2008). Stress-responsive microRNAs in *Populus*. Plant J..

[54] Chen L., Ren Y., Zhang Y., Xu J., Sun F., Zhang Z., Wang Y. (2012). Genome-wide identification and expression analysis of heat-responsive and novel microRNAs in *Populus tomentosa*. Gene.

[55] Mahale B.M., Fakrudin B., Ghosh S., Krishnaraj P.U. (2014). LNA mediated in situ hybridization of miR171 and miR397a in leaf and ambient root tissues revealed expressional homogeneity in response to shoot heat shock in *Arabidopsis thaliana*. J. Plant Biochem. Biotechnol..

[56] Guan Q., Lu X., Zeng H., Zhang Y., Zhu J. (2013). Heat stress induction of *miR398* triggers a regulatory loop that is critical for thermotolerance in Arabidopsis. Plant J..

[57] Sunkar R., Zhu J.K. (2004). Novel and stress-regulated microRNAs and other small RNAs from *arabidopsis*. Plant Cell.

[58] Beauclair L., Yu A., Bouché N. (2010). microRNA-directed cleavage and translational repression of the copper chaperone for superoxide dismutase mRNA in Arabidopsis. Plant J..

[59] Sunkar R., Kapoor A., Zhu J.K. (2006). Posttranscriptional induction of two Cu/Zn superoxide dismutase genes in *Arabidopsis* is mediated by downregulation of miR398 and important for oxidative stress tolerance. Plant Cell.

[60] Yu X., Wang H., Lu Y., de Ruiter M., Cariaso M., Prins M., van Tunen A., He Y. (2012). Identification of conserved and novel microRNAs that are responsive to heat stress in *Brassica rapa*. J. Exp. Bot..

[61] Zhang Y., Zhao G., Li Y., Mo N., Zhang J., Liang Y. (2017). Transcriptomic analysis implies that GA regulates sex expression via ethylene-dependent and ethylene-independent pathways in cucumber (*Cucumis sativus* L.). Front. Plant Sci..

[62] Zhou J., Xiong Q., Chen H., Yang C., Fan Y. (2017). Identification of the spinal expression profile of non-coding RNAs involved in neuropathic pain following spared nerve injury by sequence analysis. Front. Mol. Neurosci..

[63] Zhang S., Zhu D., Li H., Li H., Feng C., Zhang W. (2017). Characterization of circRNA-associated-ceRNA networks in a senescence-accelerated mouse prone 8 brain. Mol. Ther..

[64] Kim D., Langmead B., Salzberg S.L. (2015). HISAT: A fast spliced aligner with low memory requirements. Nat. Methods.

[65] Frazee A.C., Pertea G., Jaffe A.E., Langmead B., Salzberg S.L., Leek J.T. (2015). Ballgown bridges the gap between transcriptome assembly and expression analysis. Nat. Biotechnol..

[66] Anders S., Huber W. (2010). Differential expression analysis for sequence count data. Genome Biol..

[67] Young M.D., Wakefield M.J., Smyth G.K., Oshlack A. (2010). Gene ontology analysis for RNA-seq: Accounting for selection bias. Genome Biol..

[68] Kanehisa M., Araki M., Goto S., Hattori M., Hirakawa M., Itoh M., Katayama T., Kawashima S., Okuda S., Tokimatsu T. (2008). KEGG for linking genomes to life and the environment. Nucleic Acids Res..

[69] Mao X., Cai T., Olyarchuk J.G., Wei L. (2005). Automated genome annotation and pathway identification using the KEGG Orthology (KO) as a controlled vocabulary. Bioinformatics.

[70] Pérez-Rodríguez P., Riaño-Pachón D.M., Corrêa L.G.G., Rensing S.A., Kersten B., Mueller-Roeber B. (2010). PlnTFDB: Updated content and new features of the plant transcription factor database. Nucleic Acids Res..

[71] Salmena L., Poliseno L., Tay Y., Kats L., Pandolfi P.P. (2011). A ceRNA Hypothesis: The rosetta stone of a hidden rna language?. Cell.

[72] Tay Y., Rinn J., Pandolfi P.P. (2014). The multilayered complexity of ceRNA crosstalk and competition. Nature.

