# Supplementary figures and images for: Early Response of Radish to Heat Stress by Strand-Specific Transcriptome and miRNA Analysis
Source: Int J Mol Sci. 2019 Jul 6;20(13):3321. doi: 10.3390/ijms20133321 (PMC6651063; doi:10.3390/ijms20133321)

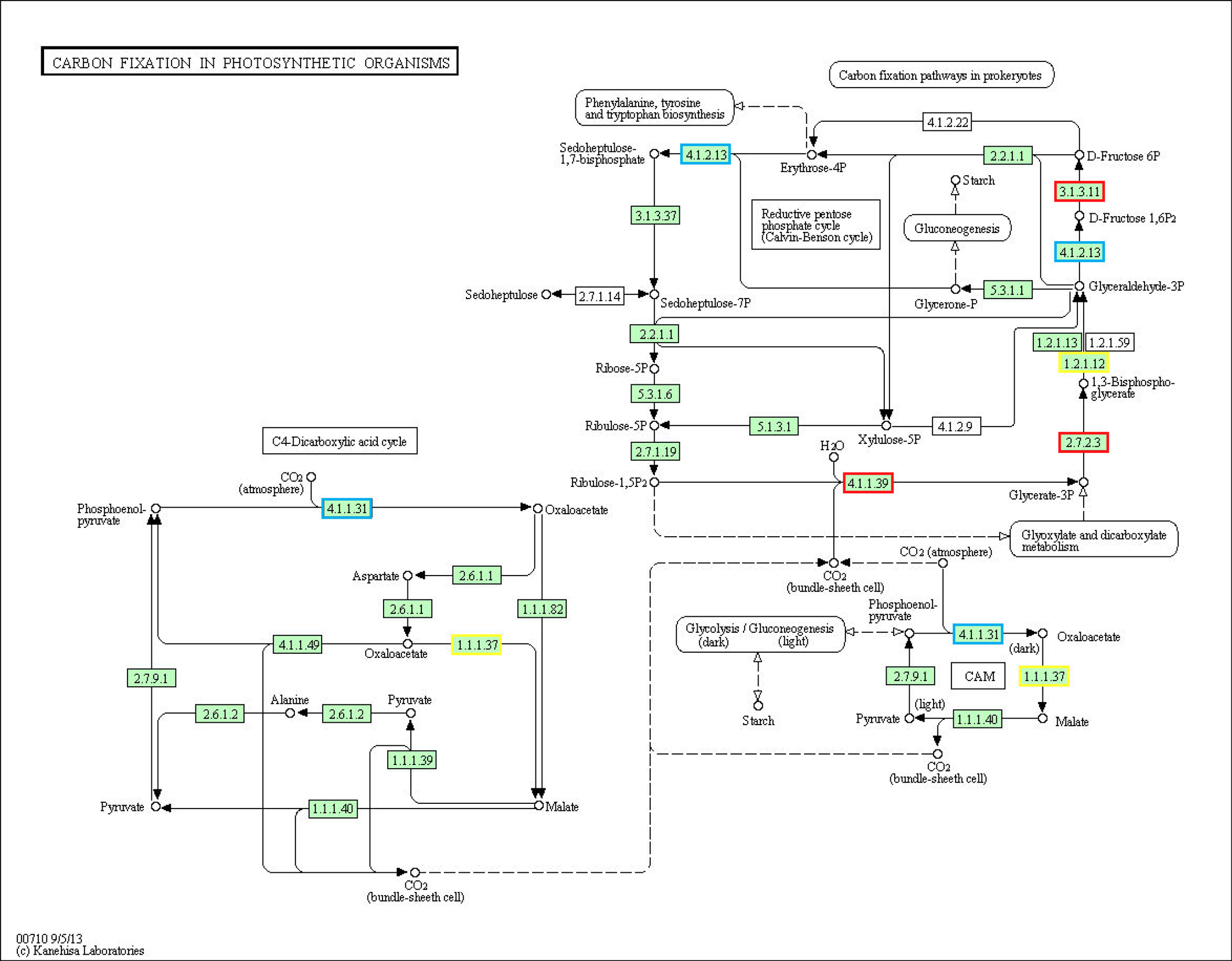

Supplement: Supplementary file 1 [file ijms-20-03321-s001.zip › Figure S1 The í░carbon fixation in photosynthetic organismsí▒ pathway enriched by KEGG analysis of DE mRNA corresponding genes.tif]

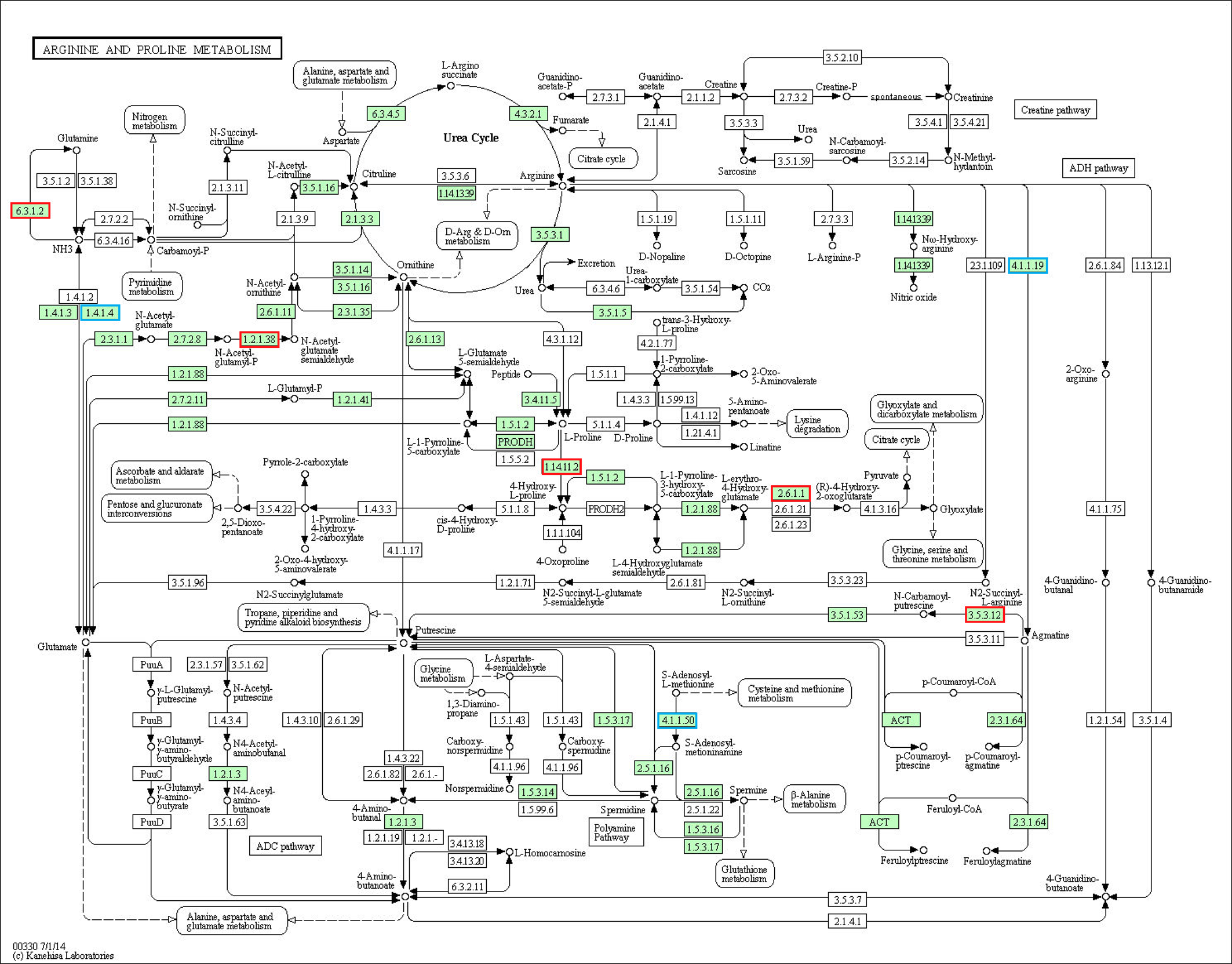

Supplement: Supplementary file 1 [file ijms-20-03321-s001.zip › Figure S2 The í░arginine and proline metabolismí░ pathway enriched by KEGG analysis of DE mRNA corresponding genes.tif]

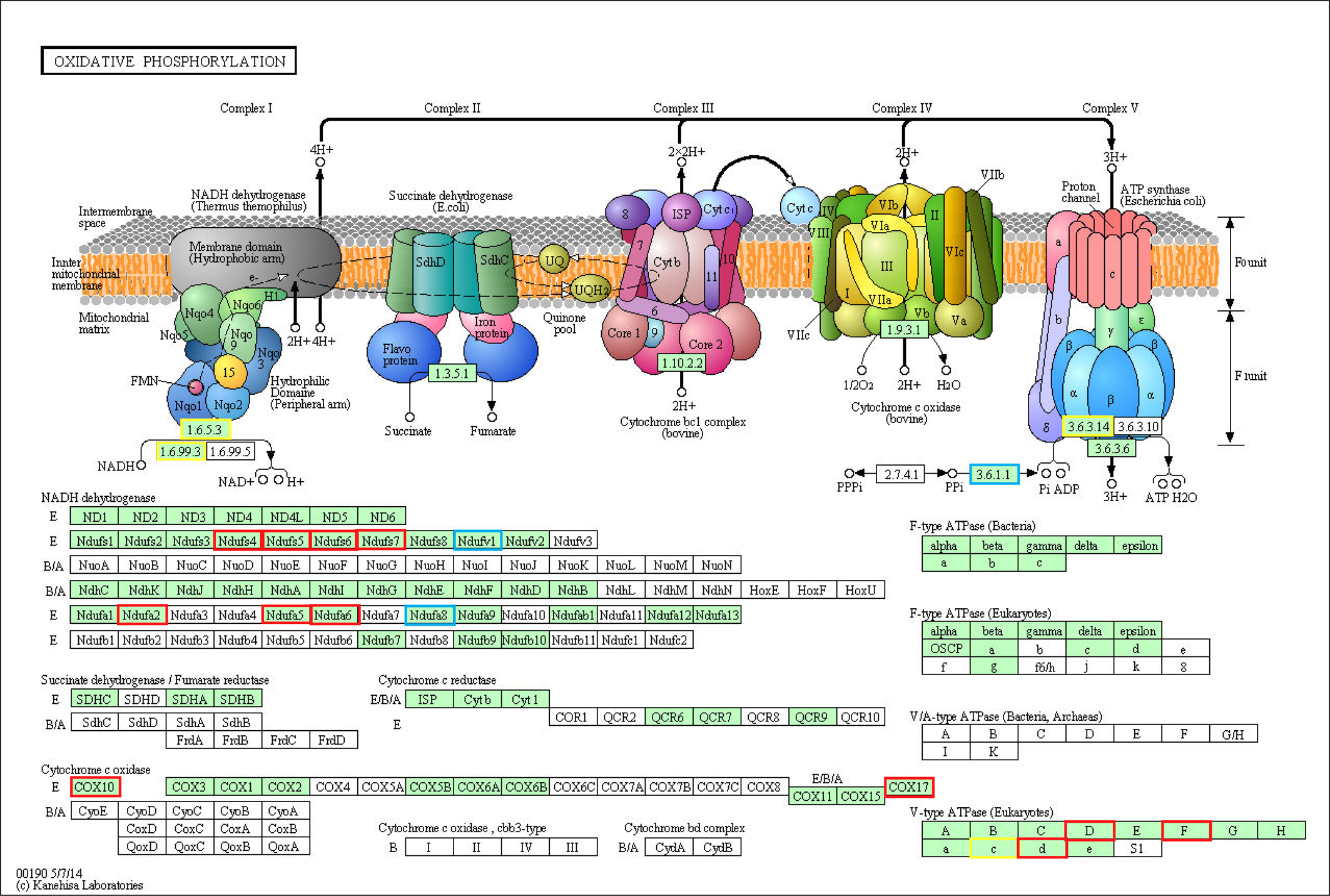

Supplement: Supplementary file 1 [file ijms-20-03321-s001.zip › Figure S3 The í░oxidative phosphorylationí▒ pathway enriched by KEGG analysis of DE mRNA corresponding genes.tif]

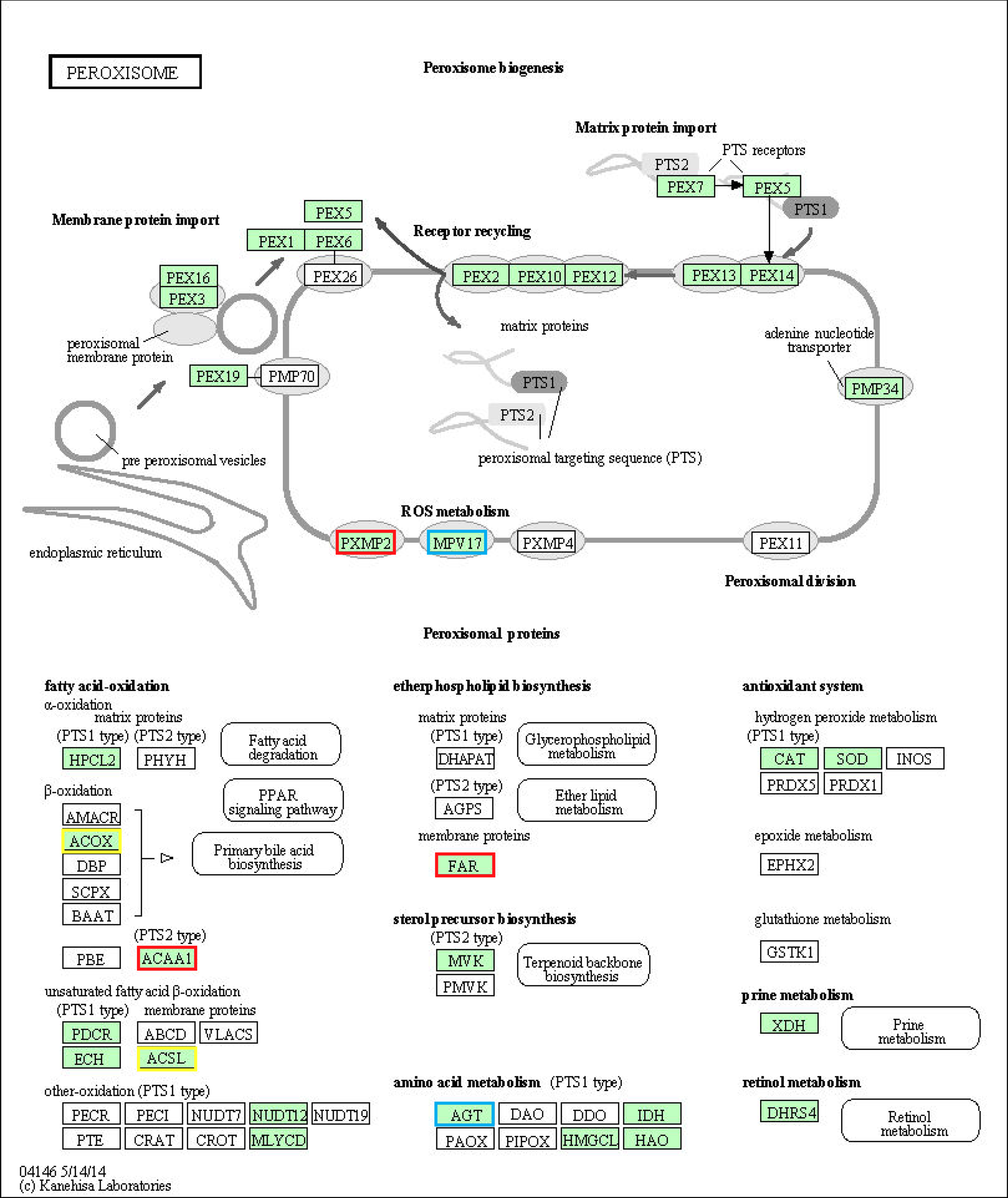

Supplement: Supplementary file 1 [file ijms-20-03321-s001.zip › Figure S4 The í░peroxisomeí▒ enriched by KEGG analysis of DE mRNA corresponding genes.tif]

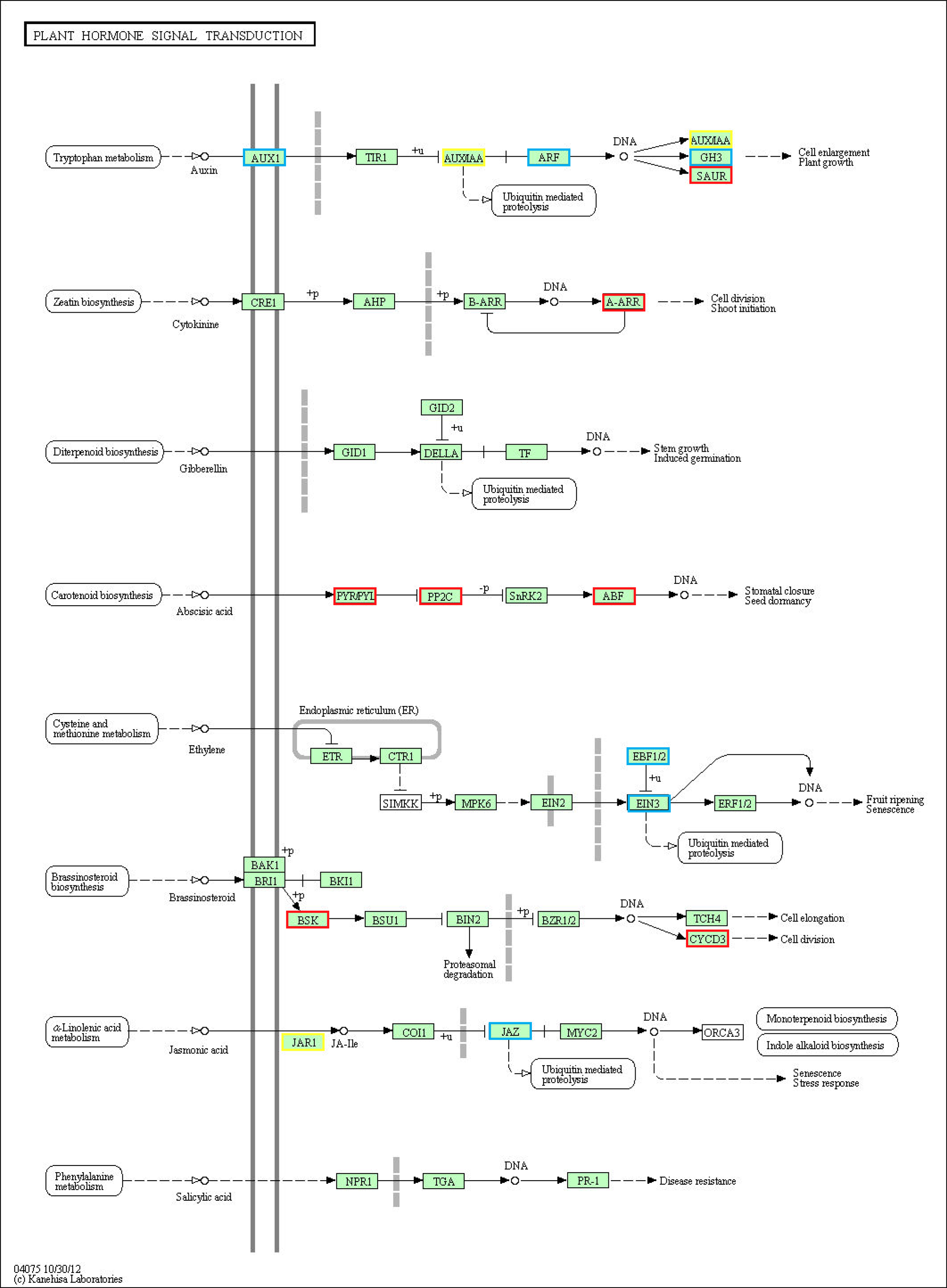

Supplement: Supplementary file 1 [file ijms-20-03321-s001.zip › Figure S5 The í░plant hormone signal transductioní▒ pathway enriched by KEGG analysis of DE mRNA corresponding genes.tif]
